# Research design and statistical methods in Pakistan Journal of Medical Sciences (PJMS)

**DOI:** 10.12669/pjms.321.9033

**Published:** 2016

**Authors:** Sohail Akhtar, Syed Wadood Ali Shah, M. Rafiq, Ajmal Khan

**Affiliations:** 1 Dr. Sohail Akhtar, PhD (UK), Department of Statistics, University of Malakand, Lower Dir, KPK, Pakistan; 2 Syed Wadood Ali Shah (PhD Candidate), Department of Pharmacy, University of Malakand, Lower Dir, KPK, Pakistan; 3 M. Rafiq (M.Phil), Department of Statistics, University of Malakand, Lower Dir, KPK, Pakistan; 4 Ajmal Khan, Department of Zoology, University of Malakand, Lower Dir, KPK, Pakistan

**Keywords:** Biostatistics, Statistical analysis, Study design, Statistical methods, Bibliometric analysis

## Abstract

**Objective:**

This article compares the study design and statistical methods used in 2005, 2010 and 2015 of Pakistan Journal of Medical Sciences (PJMS).

**Methods:**

Only original articles of PJMS were considered for the analysis. The articles were carefully reviewed for statistical methods and designs, and then recorded accordingly. The frequency of each statistical method and research design was estimated and compared with previous years.

**Results:**

A total of 429 articles were evaluated (n=74 in 2005, n=179 in 2010, n=176 in 2015) in which 171 (40%) were cross-sectional and 116 (27%) were prospective study designs. A verity of statistical methods were found in the analysis. The most frequent methods include: descriptive statistics (n=315, 73.4%), chi-square/Fisher’s exact tests (n=205, 47.8%) and student t-test (n=186, 43.4%). There was a significant increase in the use of statistical methods over time period: t-test, chi-square/Fisher’s exact test, logistic regression, epidemiological statistics, and non-parametric tests.

**Conclusion:**

This study shows that a diverse variety of statistical methods have been used in the research articles of PJMS and frequency improved from 2005 to 2015. However, descriptive statistics was the most frequent method of statistical analysis in the published articles while cross-sectional study design was common study design.

## INTRODUCTION

Nowadays, biostatistics play an extremely active role in the field of medical research to provide good services to public health. New analytical techniques and tools for biostatistics are consistently being found. There has been a tremendous growth in the development of statistical techniques and methods over the past few decades. These promising areas of application consist of medical trials, investigateive genomics, observational type study, imaging and structural study. Simply, statistics has become indispensable to evaluate treatment effects in medical research.^1^-^3^ Therefore, the appropriate use of bio-statistical techniques and methods are becoming more and more important in biomedical research. Many biomedical journals have hired statisticians to review the appropriateness of statistical methods and approaches.

During the last few years, a number of papers have been published to illustrate the use of statistical techniques and study design in biomedical journals.^4^-^17^ Most of these review papers showed that there has been a positive improvement in the use of statistical methods in medical research. Two similar studies have been published for Pakistani journals which mainly focused on the comparison of statistical methods and research designs of indexed and non-indexed journals.^18^,^19^ However, in this article, we compared the level of improvement of statistical content and research design used during three different years (2005, 2010, 2015) of Pakistan journal of Medical Sciences. Pakistan journal of Medical Sciences is a bi-monthly and ISI indexed journal. The journal has been successfully continuing to hold a broad range of articles on general medicine and health, and has been provided a significant source of medical research in Pakistan. So this study will help the biomedical readers and researchers to understand the level of statistics that have been published in Pakistan Journal of Medical Sciences.

## METHODS

To perform the analysis, all the original papers were downloaded from the PJMS web-site for these each years from those published in the year 2005, 2010 and 2015 (Jan to August). A total of 429 articles were studied. Only original research articles were included in the study and case reports, reviews, letters to the editor, editorials, systematic reviews, conference proceedings, meta-analysis, publication audit were excluded from the analysis.

Each article was carefully reviewed to draw out the research design and type of statistical methods used in their analysis. An excel spreadsheet was used to record the required information from the articles. Through the investigation, we found all the major research designs (cross-sectional survey, prospective study, retrospective study, randomized clinical trial (RCT)). On the other hand, an extensive variety of statistical methods were found in the review of PJMS articles. The statistical methods were categorized in the almost similar manner as published in earlier literature with some modification (Table-I).^4^,^20^ Initially, the statistical methods were categorized in parametric and non-parametric tests. These tests were categorized into basic and advance. In the investigation, a wide number of basic tests were found, for example, chi-square/Fisher’s exact test, student *t* -- test, analysis of variance (ANOVA), regression analysis, etc. On the other hand several advanced statistical methods were found, such as, logistic regression, receiver operating characteristic (ROC), sensitivity, specificity, odd ratio, etc. A comparison of these three years (2005, 2010, 2015) was made by calculating the frequency of each research design and statistical content used. Chi--square test was then used to check the statistical difference between these frequencies of three time periods. Statistical software SPSS version 20 was used for all calculations and value with p<0.05 was considered significant.

**Table-I:** Classification of statistical methods as used in PJMS in 2005, 2010 and 2015.

Statistical Method	Description
Descriptive statistics	Ratios and percentages means, standard deviations, standard errors.
Student t-tests	One-sample, two-samples t-tests and paired samples
Contingency tables	Chi-square (χ^2^) tests, Fisher’s exact test, McNemar’s test
Parametric Correlation	Pearson correlation coefficient,
linear regression	Least squares regression with one outcome variable and one or more predictor variables
Epidemiological statistics	Statistics relative risk, odds ratio, log odds, measures of association, sensitivity, specificity, receiver operating characteristic (ROC)
Multi-way tables	log-linear models
Non-parametric test	Sign test, Wilcoxon signed-rank test, Mann-Whitney test, Kruskal-Wallis Test, Friedman Test, Shapiro-Wilk test
Non-parametric Correlation	Kendall rank, Spearman
Analysis of variance	Analysis of variance (ANOVA), analysis of co-variance (ANCOVA)
Multiple comparisons	To find out the source of the significant differences among the group means: Dunnett’s procedure, Tukey’s Method, Fisher’s LSD procedure.
Regression for survival	Includes logistic regression, Poisson regression
Sensitivity analysis	Kaplan–Meier method, log-rank test
Other	Anything not fitting above headings, Factor analysis, Bartlett’s Test, Shapiro-Wilk’s

## RESULTS

A total of 429 articles were studied for this investigation: 74 (17.2%) from 2005, 179 (41.7%) from 2010, and 176 (41.0%) from 2015. Majority of the articles used a cross-sectional study design (n=171, 40%), prospective study design (n=116, 27%), retrospective study (n=78, 18%), randomized clinical trials (n=34, 8%). There were no significant differences (with p=0.055) in study designs used over the three periods (2005, 2010, 2015). We also noted that (n=30, 7%) manuscripts did not mention a very clear research design.

A number of statistical methods were found during the review of published articles (Table-III). Majority of the articles used descriptive statistics: 50 (67.6%) from 2005, 135 (75.4%) from 2010 and 130 (74.0) from 2015. The p--value (0.430) shows that the trend of descriptive statistics was insignificant over the three periods at 5% level of significance. The trend of the use of correlation, regression and analysis of variance were slightly greater in percentage over time periods but not statistically significant. On the other hand there was a significant increase in the use of statistical methods: student *t*-- test (48.9% in 2015 vs. 27.0% in 2005), logistic regression (9.7% in 2015 vs 5.4% in 2005), chi-- square/Fisher’s exact test (51.1% in 2015 vs. 20.3% in 2005), epidemiological statistics (17.6% in 2015 vs. 4.1 in 2005), non parametric tests (24.4% in 2015 vs 5.4% in 2005).

**Table-II:** Summary of research design used in the samples.

	2005		2010		2015 (Jan. to Aug.)		P-Value

	Articles	%	Articles	%	Articles	%	
Cross-sectional survey	28	38	75	42	68	39	0.055
Retrospective study	10	14	30	17	38	22	
Prospective study	18	24	47	26	51	29	
Randomized controlled trials	6	8	17	9	11	6	
Not clear from study	12	16	10	6	8	5	
	74		179		176		

*chi-square statistic is 15.227. The *p*-value is 0.055. The result is not significant at p < 0.05.

**Table-III:** Outline of statistical methods used in PJMS.

Statistical methods	2005		2010		2015 (Jan. to Aug.)		p-value

	n=74	%	n=179	%	n=176	%	
Descriptive statistics	50	67.6	135	75.4	130	74.0	0.430
t-- test (independent/paired)	20	27.0	80	44.7	86	48.9	0.005
Logistic regression	4	5.4	10	5.6	17	9.7	0.030
Regression	2	2.7	4	2.2	10	5.7	0.202
ANOVA/ANCOVA	3	4.1	16	8.9	24	13.6	0.058
Correlation	10	13.5	20	11.2	21	11.9	0.872
Contingency table (Chi-square/Fisher’s exact)	15	20.3	100	55.9	90	51.1	0.000
Epidemiological statistics	3	4.1	14	7.8	31	17.6	0.001
Nonparametric	4	5.4	17	9.5	43	24.4	0.000

A number of other different statistical methods were reported only once or twice in the PJMS articles, for example, factor analysis, component analysis, and Poisson regression, etc (see Table-II). Therefore, we ignored these statistical methods in the Table-III.

## DISCUSSION

Our findings show that cross-sectional research design was the most common study in the published articles of PJMS during each of the three years. This result is consistent with other published studies.^21^,^22^ Prospective study designs is used in almost one third of the published articles and is the second most frequent type of research design. On the other hand, there is a low frequency of retrospective study and randomized clinical trials in the published articles. Furthermore, there was no statistical significance difference between research designs used over three times periods.

Previous reports shows that descriptive statistics is the most frequent method of analysis in publications of PJMS.^23^ Simple statistical methods like student *t* -- tests, correlation, chi-- square and Fisher’s exact tests, Mann--Whitney, Kruskal--Wallis and Wilcoxon signed rank test are frequently used. There is a significant increase in the number of articles using chi--square and *t* -- tests between the three periods. This is consistent to previously published studies.^23^ We also found that there was a significant increase in the reports using chi-square, *t*–test, epidemiological statistics and logistic regression between the three time periods. However, the frequency of complex statistical methods like logistic regression, Poisson regression and factor analysis is quite low as compared to simple statistical methods

## CONCLUSION

In conclusion, the current analysis shows that Pakistan Journal of Medical Sciences (PJMS) used all research study designs in published articles but there is still much room for progress concerning advance statistical methods. Most of the published articles continue to use a cross-sectional design while retrospective study and randomized clinical trials still require more attention as compared to journals of advanced countries. It is noted that a variety of statistical methods were found in the published articles but the frequency of advance statistical methods was quite low as compared to advanced countries journals. Furthermore, it is concluded that the medical researchers and readers should have at least the basic knowledge of biostatistics to understand the biomedical research and its interpretation.
